# Perception of Healthcare Providers About the Use of Social Media to Manage a Healthy Diet in Saudi Arabia

**DOI:** 10.3389/fpubh.2021.543913

**Published:** 2021-06-14

**Authors:** Turki Alanzi, Maryam Altuwailib, Amjad Mohammed Saadah, Fahad Alanezi

**Affiliations:** ^1^Health Information Management and Technology Department, College of Public Health, Imam Abdulrahman Bin Faisal University, Dammam, Saudi Arabia; ^2^Community College, Imam Abdulrahman Bin Faisal University, Dammam, Saudi Arabia

**Keywords:** healthcare, food diet, Saudi Arabia, perception, manage, social networks

## Abstract

**Purpose:** The objective of this study is to investigate the perceptions of healthcare providers about the use of social media for healthy diet management in Saudi Arabia.

**Participants and Methods:** A cross-sectional study was designed to investigate the use of social media healthy diet management. The sample constituted 308 healthcare professionals from Saudi Arabia. The social media application, WhatsApp is employed to distribute the questionnaire, which has achieved a response rate of 50.61%. Out of the total participants, 55% of the participants were under 30 years of age, 71% were females, and 55% of the participants had a bachelor's degree.

**Results:** Among the total respondents, 66% used social networking applications more than four hours a day, and 78% utilized social media to get information about a healthy diet. The respondents employed the following platforms for this purpose: Instagram (27%), YouTube (19%), Snapchat (19%), WhatsApp (18%), Twitter (8%), and Facebook (5%). The respondents considered that social media is very helpful to educate (44%), communicate with specialists (33%), and get applications for a healthy diet (38%). They held an opinion that social media is very helpful in improving knowledge about a healthy diet (47%), creating diet awareness (42%), and achieving healthy diet outcomes (37%) and lifestyle (37%).

**Conclusion:** According to the perception of health providers, social media can be used to promote healthy diet management in Saudi Arabia. Also, the growing use of social media in Saudi Arabia represents the potential to create programs that encourage and promote healthy eating habits in the Kingdom of Saudi Arabia. Instagram, YouTube, Snapchat, and WhatsApp platforms can be used for this purpose.

## Introduction

In general, social media platforms display articles, advertisements, videos, blogs, and various types of information related to nutrition, food, beverages, recipes, diets, physical exercise, and other issues related to the search for a healthy lifestyle (1, 2).

In addition, these tools are increasingly used to provide information on nutrition, food, and health promotion interventions (3–5). These interventions have been directed to promote a healthy diet in obese and overweight people, patients with type 1 and type 2 diabetes, patients with metabolic syndromes, and people with other diseases (5–8).

Obviously, a diet with healthy habits combined with physical exercise, that is, a behavioral diet, leads to the prevention of diseases and promotes a good state of physical and mental health and well-being. On the contrary, an unbalanced diet without healthy dietary habits and without fiscal exercise can lead to various health issues, such as obesity, being overweight, cardiovascular diseases, diabetes, cancer, metabolic disorders, and other health problems (5).

It is convenient to indicate that the use of social media to disseminate knowledge and exchange information on nutritional topics increases every day, but its use is still incipient (4, 6). As identified in few studies, social networks can play an important role for people to manage a healthy diet that contributes to maintaining a good state of health (1–3, 9). Also, in a survey conducted in Australia, most dietitians used social media for professional development and networking, with Facebook and Instagram being the most commonly used platforms (6). Likewise, social media platforms have been used to distribute information on nutrition education (10).

On the other hand, it is convenient to point out that according to several studies, part of the information published on social media is not accurate and does not allow achieving a healthy diet. (1, 11–13). Similarly, some authors who publish articles in social media lack scientific and professional information in the field of healthy and balanced nutrition (11).

In relation to Saudi Arabia, a national survey with 10,735 participants revealed that a high percentage of the population did not have a healthy diet, indicating that it is necessary to develop programs to promote healthy eating habits and avoid diseases caused by poor diet (14). Additionally, another survey conducted with a group of young Saudi adults found similar results. (15). In addition, the extent of false news, and issues such as privacy and security, may affect the reliability on social media for health information dissemination and awareness creation (16, 17). Moreover, it was identified that young adults were more inclined toward the use of social media for their diet management, compared to older people (18). In relation to the gender, there is a significant difference between the genders in terms of social media use for dietary management (19, 20). In a recent study, it was identified that females demonstrated higher vegetable consumption in the daily diet and greater satisfaction in the educational context compared to males (21).

Parallel with this situation, the use of social networks in Saudi Arabia has increased progressively in recent years, impacting the different activities carried out in this country (22). As of January 2021, there are 33.58 M internet users representing 95.7% of Saudi Arabian population with 27.8 million active social media users representing 79.3% of total population. These statistics represent a huge potential for social media in disseminating healthcare services and creating health awareness. In addition, there is an increasing reliance on online and social media channels for health information management due to the Covid-19 pandemic, as a result of which there is a growing need for research in the application of social media in health information management (23).

Accordingly, the research studies in Saudi Arabia focused on the social media applications in health management from different perspectives. For instance, Aldossari and Al-Mahish study (24) focused on the used social media preferences for determining the food habits of the Saudi Arabian residents and found that the majority of the people preferred unhealthy and nutrient-poor food items over healthy and nutrient-rich food. Similarly, Al-Hamdan et al. (25) investigated the use of social media (WhatsApp) as an intervention for educating and creating health awareness among Saudi females with prediabetes and found that the application was significant in creating awareness and creating behavioral change, resulting in improved HbA1c levels. Similarly, another study (26) has identified that social media applications were the preferred sources for searching health-related information among the pregnant women. These studies focused on different perspectives of the influence of social media in health management. However, there is a lack of research on investigating the social media in creating awareness about healthy diet management and practices. Moreover, there is an increasing concern over the reliability of healthcare, dietary, and lifestyle information on the social media platforms, as many of these “influencers” on social media platforms may be poorly qualified in order to provide nutritional or dietary guidance, and advice given may be without accepted scientific evidence and contrary to public health policy (27). This development of social networks in Saudi Arabia suggests that there is a potential to use these tools in order to advance programs to help people improve their eating habits. In this regard, it should be noted that there has been no research on this subject in Saudi Arabia.

Therefore, the objective of this study is formulated to investigate the perceptions of healthcare providers about the use of social media to manage a healthy diet in Saudi Arabia.

## Methods

### Study Settings and Participants

In this research, a cross-sectional survey is designed to investigate the use of social media to manage a healthy diet. The sample population constituted 308 healthcare professionals from the Kingdom of Saudi Arabia. A response rate of 50.61% was achieved. The informed consent from the participants was gathered before the survey. Ethical approval was provided by the Abdulrahman Bin Faisal University, Saudi Arabia.

### Inclusion and Exclusion Criteria

All healthcare providers living in Saudi Arabia were included in this study.

### Data Collection

The questionnaire survey was distributed to the participants using WhatsApp and other social media platforms, and the data was collected in January 2019.

### Questionnaire Design

The questionnaire used in the survey was designed by the research team and contained nine items that pointed to the collection of data relating to the main objective of the study (28). The questions were generally closed-ended and the participants responded to them by choosing among multiple-choice answers. At the beginning of the questionnaire, the survey instructions and an explanatory introduction to indicate the purpose of the survey was included. Also, an appreciation for participation in the study was offered. The questionnaire questions were tested before the actual use with the participants, and it was established that they were clear and unambiguous. It was also found that the questions were understood and did not generate faulty or hostile answers.

The first four items were aimed at obtaining demographic information of the participants: age, gender, level of education, and professional specialty. Item 5 asked about the number of hours that participants used social media. Item 6 inquired whether participants obtained information about a healthy diet through social media (Yes-No). Item 7 examined the type of social media used by the participants to obtain information about a healthy diet (Facebook, Twitter, Instagram, YouTube, Snapchat, WhatsApp, other). Item 8 contained several questions with the intention of evaluating the utility of social media as a tool to achieve a healthy diet (education about healthy diet, awareness about healthy diet, knowledge about healthy diet, improvement of health outcomes, communication with nutrition specialist, finding out applications to develop a healthy diet, influence of social media information on lifestyle); these questions were evaluated using the scale: very helpful, some helpful, not helpful, not applicable. Finally, item 9 asked the question, Do you think that social media helps you to reach a healthy behavioral diet and encourage people for a healthy diet? (Yes- No).

### Statistical Analysis

Standard computation methods were used in calculating basic descriptive statistics. The data were expressed in percentages, and the mean (M), the standard deviation (SD), and the confidence interval (CI) 95% were estimated and are presented in the tables and figures of the Results section.

## Results

The demographical information of the healthcare providers surveyed in this study is shown in Table 1. In this table, we observe that more than half of the participants (55%) were under 30 years of age, and most of the respondents were females (71%). Also, 47% of the respondents were physicians, nurses, pharmacists, and professionals from the HIMT-management quality field, and the rest of them (53%) belonged to other healthcare areas. As well, more than half of the participants (55%) had a bachelor's degree.

**Table 1:** Demographic information of the participants (*n* = 308).

	***n* (%)**	**M**	**SD**	**CI**
**Age** (years)		1.63	0.846	1.53–1.72
20-30	170 (55)			
31-40	103 (33)			
41-50	17 (6)			
>50	18 (6)			
**Gender**		1.71	0.454	1.66–1.70
Male	89 (29)			
Female	219 (71)			
**Educational level**		2.38	1.16	2.25–2.51
Diploma	53 (17)			
Bachelor	170 (55)			
Master	35 (11)			
Doctorate	18 (6)			
Other	33 (11)			
**Professional specialty**		3.62	1.584	3.44–3.79
Physician	35 (11)			
Nurse	81 (26)			
Pharmacist	15 (5)			
HIMT(Management quality)	14 (4)			
Other	164 (53)			

In relation to the daily use of social media, Table 2 indicates that more than half of the population (66%) used social networks more than 4 h a day.

**Table 2:** Daily usage of social media (*n* = 308).

	**%**	**M**	**SD**	**CI**
**Time** (hours)		2.84	0.909	2.74–2.95
<1	7			
1–2	27			
3–4	39			
>5	27			

**Figure 1:**
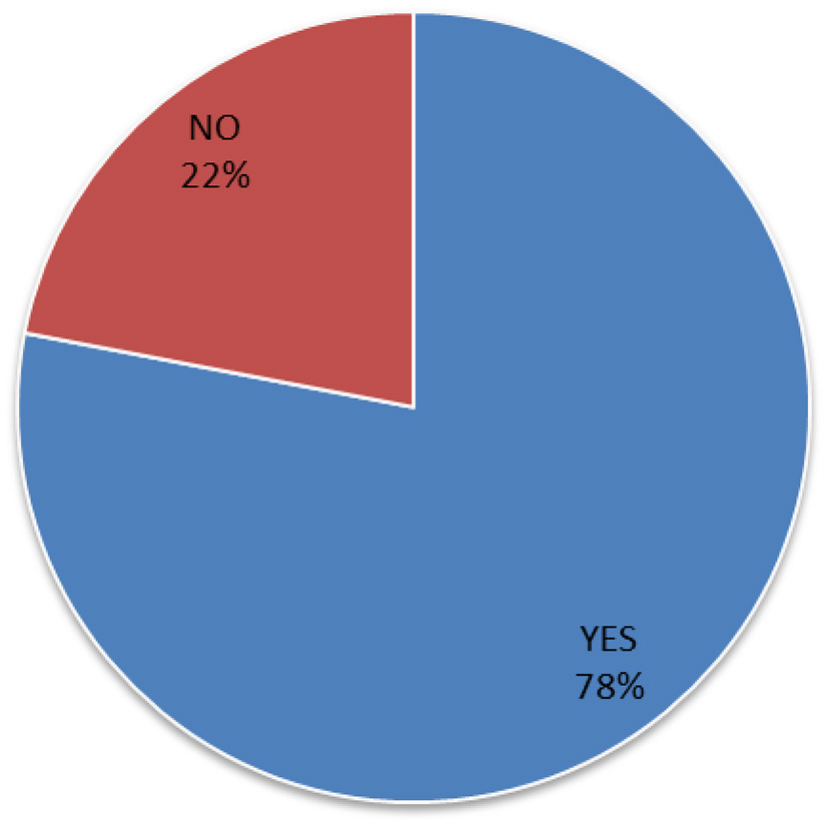
Percentage of participants that get information about a healthy diet in social media (*n* = 308, M = 1.22, SD = 0.41, CI = 1.17–1.27).

Also, in Figure 1 we detect that 78% of the participants utilized social networks to get information about a healthy diet.

**Figure 2:**
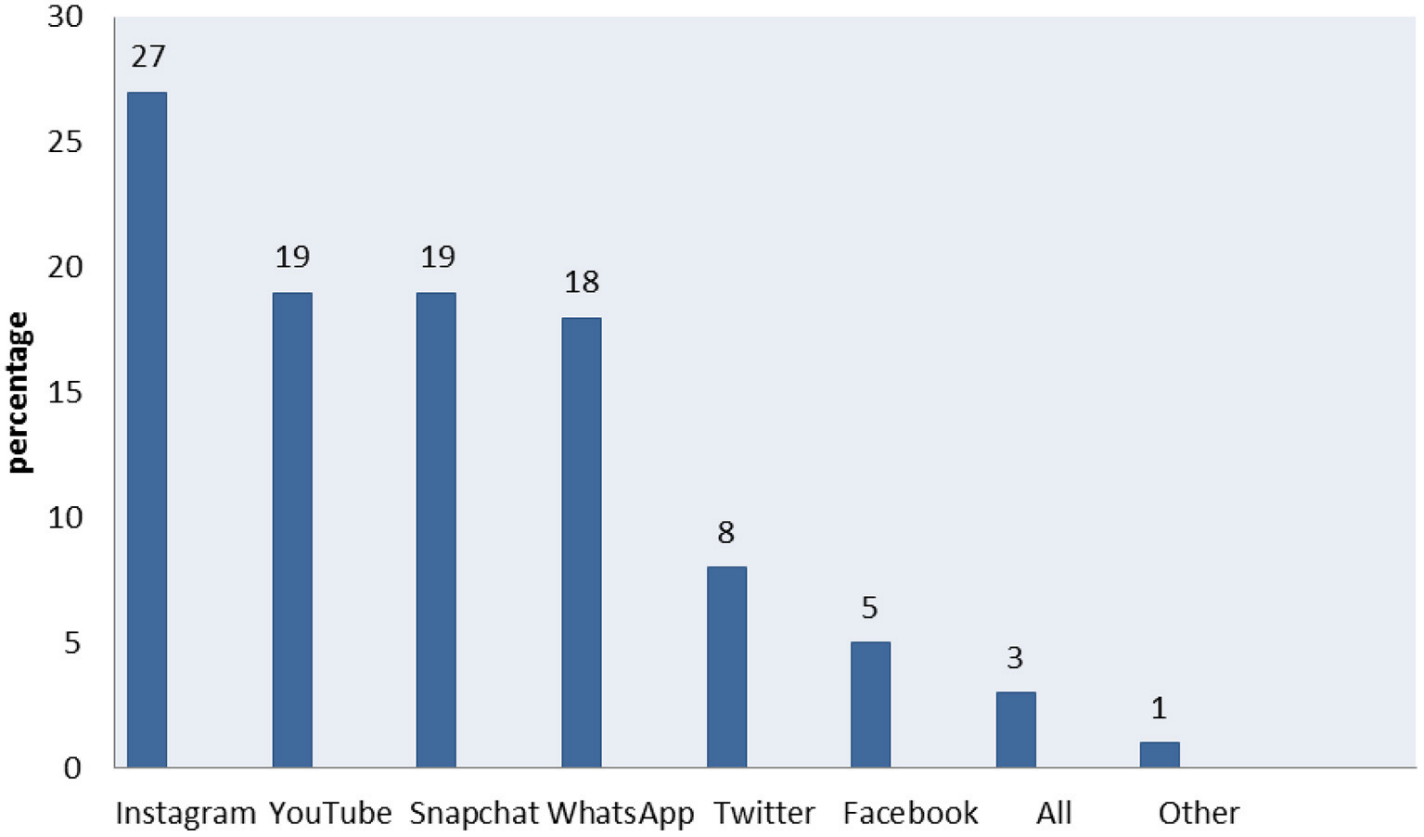
Types of social media used to find information about a healthy diet (*n* = 308, M = 4.11, SD = 1.534, CI = 3.97–4.24).

In addition, according to Figure 2, the respondents employed the following social media platforms for this purpose: Instagram (27%), YouTube (19%), Snapchat (19%), WhatsApp (18%), Twitter (8%), Facebook (5%), a combination of all the above (3%), and other sites (1%).

On the other hand, in Table 3 is expressed the opinion of the participants related to the utility of social media to manage a healthy diet. The usefulness of social media is evaluated using the scale: very helpful, some helpful, not helpful, and not applicable. Here is considered the use of social media to improve education, knowledge, awareness, and outcomes about a healthy diet. Also, it is assessed the helpfulness of these tools to communicate with nutrition specialists, to get applications for developing a healthy diet, and to improve lifestyle.

**Table 3:** Usefulness of social media to manage a healthy diet (*n* = 308).

	**Very helpful**	**Somewhat helpful**	**Not helpful**	**Not applicable**
	**%**	**%**	**%**	**%**
A tool to educate about a healthy diet	44	50	4	2
An instrument to improve knowledge about a healthy diet	47	43	9	1
A practice to enhance healthy diet outcomes	37	50	10	3
A way to increase healthy diet awareness	42	50	6	2
A system to communicate with nutrition specialists	33	42	15	10
A system to get applications to develop a healthy diet	38	43	11	8
An alternative to improve lifestyle	37	52	8	3

**Figure 3:**
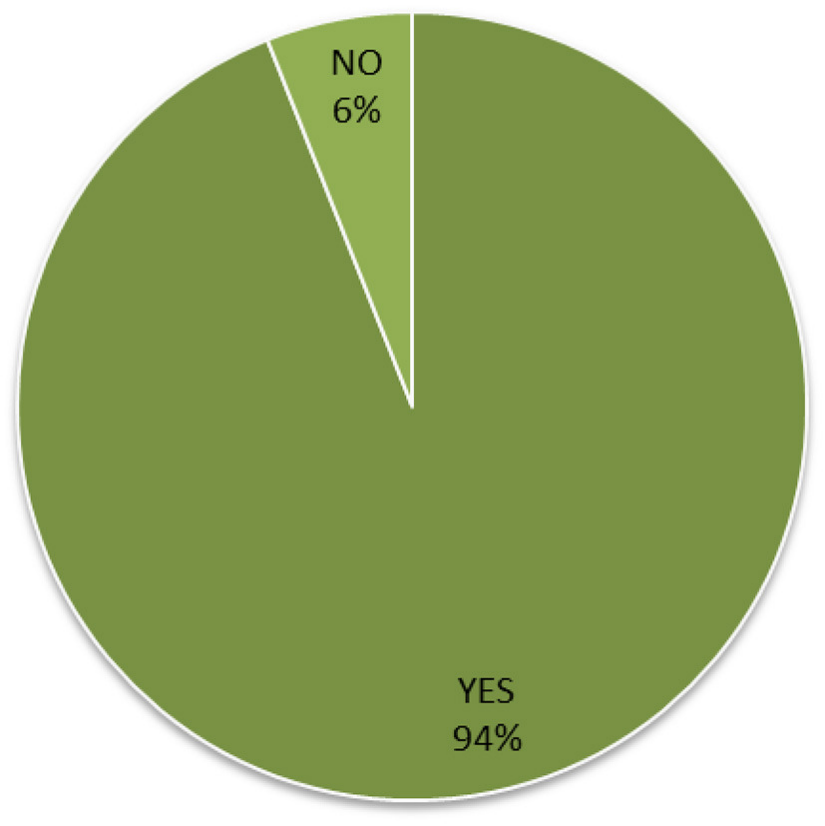
Role of social media to encourage and manage a healthy diet (*n* = 308, M = 1.06, SD = 0.243, CI = 1.03–1.09).

Lastly, Figure 3 shows that 94% of the surveyed healthcare providers expressed that social media platforms help them to encourage and manage a healthy diet.

## Discussion

The results of this study related to the perception of health providers to manage a healthy diet in Saudi Arabia indicated that most participants considered that social media was useful for this purpose.

In this sense, in Figure 1 it was observed that a high percentage of participants (78%) used social media to search for information about a healthy diet. Also, the data in Figure 3 showed that almost all health providers surveyed (94%) agreed that the information available on social media platforms encouraged them to manage and achieve a healthy diet. These observations coincide with the results of some studies that have pointed out the utility of social media to obtain adequate information on a healthy diet that contributes to maintaining a good state of health (29, 30). In this context, Arnold noted that social media is useful to get advice on a healthy diet (18). In addition, Kattelmann indicated that the use of social media encourages young adults to physical activity and healthy food habits (31). As shown in Table 3, there are few negative responses toward the use of social media. A total of 25% of the participants negatively responded (15% stated not helpful and 10% stated not applicable) about using social media to communicate with nutrition specialists. Similarly, 11% of the participants stated that social media is not helpful to get applications to develop a healthy diet. In addition, as shown in Figure 1, 22% of the total participants stated that they do not get information about a healthy diet from social media. These negative responses can be related to the reliability issues, affecting their use of social media applications (32, 33).

The findings in this study suggested that young people aged <30 years prefer social media and use it extensively for healthy diet management in similar to the study conducted by Klassen et al. (18). In addition, differences in the preferences of male and female participants were identified in this study, where female participants were more influenced by the use of social media for healthy diet management compared to males. These findings are similar to the studies conducted by Rambaree et al. (20) and Boraita et al. (21).

From Table 3, it can be interpreted that a high proportion of the sample population considered that social media networks were very helpful to educate (44%), communicate with nutrition specialists (33%), and get applications for developing a healthy diet (38%). Similarly, the majority of them thought that social media was useful in improving knowledge about a healthy diet (47%), creating health diet awareness (42%), achieving healthy diet outcomes (37%) and lifestyle (37%). With regard to these comments, Dumas et al., in an extensive review of the literature, have suggested that social media contribute to the education, translation, dissemination, and exchange of knowledge about diet and other nutritional topics (6). Also, in the aforementioned review, it is observed that, in several cases, using interventions through social media, people's health and lifestyle were improved by providing information about a healthy diet (6). In addition, Capplette et al. have shown that social media can help promote a healthy diet based on fruits and vegetables in a group of young women (5). Also, Coughlin et al. considered that in the use of applications, Smartphones and social media can be useful to increase awareness, knowledge, and education about a healthy diet (31).

On the other hand, the most used social media platform to obtain information about a healthy diet are in descending order: Instagram, YouTube, Snapchat, WhatsApp, Twitter, and Facebook. In this regard, in previous studies, it has been found that Instagram was one of the most-used platforms for disseminating information related to food and diets (32, 33).

Low sample size was identified as the main limitations of this study and the fact that some of the participants did not answer all the questionnaire questions, which resulted in a low response rate. Therefore, future studies should aim to overcome these limitations and develop programs to encourage the population to improve their eating habits using social media for this purpose.

It is pertinent to comment that our study is the first research conducted in Saudi Arabia with the intention of knowing the opinion of health providers about the use of social media to achieve healthy diet management.

## Conclusion

The perceptions of the majority of the surveyed healthcare providers indicated that social media can be useful for healthy diet management among the population of Saudi Arabia, enabling them to maintain a good state of health and well-being. In addition, the increasing use of social networks in this region suggested that there is a huge potential for integrating educational and social programs with social media applications for creating awareness to encourage and promote healthy eating habits in the Kingdom of Saudi Arabia. Considering the most frequently used platforms, Instagram, YouTube, Snapchat, and WhatsApp platforms may be the best options for creating awareness about healthy diet management. The findings in this study can have both practical and theoretical implications. First, the findings contributed to the lack of research in the application of social media for healthy diet management in Saudi Arabia through the lens of healthcare practitioners. Second, the findings can be used by the healthcare authorities in selecting the social media types and formulating the promotional and awareness plans for maintaining healthy lifestyles and adopting healthy diets among the Saudi Arabian population. However, there are few limitations that need to be considered while interpreting the results of the present study. First, a low sample population makes it hard to generalize the results. Therefore, results should be generalized with care. Second, dependency on single method (survey) for data collection may lead to a collection of limited data. As the study focuses on the use of social media, it may be necessary to analyze influence of behaviors and attitudes, for which qualitative methods such as interviews may be effective. The future research in this context can focus on collecting the perspectives of the general population on the use of social media for healthy diet management, increase sample size in the survey, and adopt mixed methods such as survey and interviews for collecting data from different samples for analysis from various perspectives.

## Data Availability Statement

The raw data supporting the conclusions of this article will be made available by the authors, without undue reservation.

## Ethics Statement

The studies involving human participants were reviewed and approved by The Institutional Review Board of the Imam Abdulrahman Bin Faisal University. The patients/participants provided their written informed consent to participate in this study.

## Author Contributions

All authors listed have made a substantial, direct and intellectual contribution to the work, and approved it for publication.

## Conflict of Interest

The authors declare that the research was conducted in the absence of any commercial or financial relationships that could be construed as a potential conflict of interest.
